# Running Velocity Does Not Influence Lower Limb Mechanical Asymmetry

**DOI:** 10.3389/fspor.2019.00036

**Published:** 2019-09-24

**Authors:** Olivier Girard, Jean-Benoit Morin, Joong Ryu, Paul Read, Nathan Townsend

**Affiliations:** ^1^Murdoch Applied Sports Science Laboratory, Murdoch University, Perth, WA, Australia; ^2^Athlete Health and Performance Research Centre, Aspetar Orthopaedic and Sports Medicine Hospital, Doha, Qatar; ^3^Université Côte d'Azur, LAMHESS, Nice, France; ^4^Aspire Academy, Doha, Qatar

**Keywords:** imbalance, symmetry angle scores, running velocity, kinetics, kinematics, spring-mass model

## Abstract

We examined the effect of running velocity upon magnitude and range of asymmetry in the main kinetics and kinematics of treadmill running at constant, submaximal velocities. Nine well-trained, un-injured distance runners ran, in a random order, at seven running velocities (10, 12.5, 15, 17.5, 20, 22.5, and 25 km.h^−1^) for 60 s (separated by > 90 s of rest) on an instrumented treadmill (ADAL3D-WR, Medical Development, France). Continuous measurement (1,000 Hz) of spatio-temporal, horizontal force production, and spring-mass characteristics was performed and data over 10 consecutive steps (5 right and 5 leg foot contacts after ~50 s of running) were used for subsequent comparisons. Group mean and the range of asymmetry scores were assessed from the “symmetry angle” (SA) formulae where a score of 0%/100% indicates perfect symmetry/asymmetry. Mean SA scores for spatio-temporal variables were lower than 2%: contact time (0.6 ± 0.1%; range: 0.4–0.7%), aerial time (1.7 ± 0.2%; range: 1.3–2.1%) as well as step length and step frequency (0.7 ± 0.2%; range: 0.5–0.9%). Mean loading rate (5.3 ± 1.1%; range: 4.1–6.9%) and spring mass model [peak vertical force: 3.2 ± 1.6% (range: 2.9–3.4%); maximal downward vertical displacement: 11.2 ± 6.0% (range: 9.2–14.0%); leg compression: 3.6 ± 1.9% (range: 2.9–5.6%); vertical stiffness: 8.8 ± 1.9% (range: 7.1–11.6%); leg stiffness: 1.6 ± 0.6% (range: 1.2–2.9%)] presented larger mean SA values. Mean SA scores ranged 1–4% for duration of braking (1.3 ± 0.3%; range: 0.9–2.0%) and push-off (1.6 ± 0.9%; range: 1.2–2.4%) phases, peak braking (2.4 ± 1.1%; range: 1.6–3.6%), and push-off (1.7 ± 0.9%; range: 1.2–2.2%) forces as well as braking (3.7 ± 2.0%; range: 2.8–5.8%) and push-off (2.1 ± 0.8%; range: 1.3–2.6%) impulses. However, with the exception of braking impulse (*P* = 0.005), there was no influence of running velocity on asymmetry scores for any of the mechanical variables studied (0.118<P<0.920). Modifying treadmill belt velocity between 10 and 25 km.h^−1^ induced large adjustments in most running kinetics and kinematics. However, there was no noticeable difference in group mean and the range of asymmetry values across running velocities, with the magnitude of these scores being largely dependent on the biomechanical variable of interest. Finally, the relatively large range of asymmetry between participants for some variables reinforces the importance of assessing asymmetry on an individual basis.

## Introduction

Completely symmetrical gait is not possible since having a dominant leg, for instance, is natural. Anecdotally, even the fastest sprinter officially recorded (Longman, 2017) may have an asymmetrical running gait, since he strikes the ground with apparently more force with his right leg than he does with his left (New York Times website). To date, a growing body of research focuses on between-leg similarities—i.e., typically measured using symmetry scores to examine their effects on athletic performance (Bishop et al., 2018). Subtle asymmetries are not always noticeable to the naked eye, even in expert athletic coaches, or necessarily kinesthetically apparent to an athlete. An advanced analysis of an individual's running pattern is therefore necessary to evaluate symmetry in biomechanical factors not readily detectable by a coach, such as ground reaction force variables.

Asymmetry occurs when there is any deviation from symmetry, i.e., the exact replication of one limb's movement by the other (Exell et al., 2012a). In the literature, comparisons of commonly used symmetry indices indicate that none is preferred, for instance, to examine the success of rehabilitation process (Błazkiewicz et al., 2014). That said, Bishop et al. (2016) argued that reporting asymmetries via the “Symmetry Angle” (SA) (Zifchock et al., 2008) method holds some advantages over other options (i.e., limb symmetry index, bilateral strength asymmetry, asymmetry index). The SA is a dimensionless measure of asymmetry that does not suffer from artificial inflation, unlike the symmetry index that requires a reference value (Exell et al., 2012a), and is therefore a robust measure of asymmetry that can be used across kinematic and kinetic variables (Carpes et al., 2010). Marked asymmetry in maximal plantar force (measured using an in-shoe pressure system), but not contact times, slightly increases with increasing treadmill velocity between 12 and 16 km.h^−1^ for athletes <9 months post anterior cruciate ligament reconstruction despite having completed functional return to sport criteria (Thomson et al., 2018). Whether the right and left legs typically apply equal ground forces (as measured directly) from a wider range of constant, slow-to-fast running velocities in apparently healthy runners remains unclear.

Monitoring for inter-limb discrepancies is becoming common practice using instrumented treadmills that allows a large sample of successive ground reaction force traces to be collected (Carpes et al., 2010). Vertical force parameters (and derived spring-mass variables) have been used to investigate potential asymmetries in stride mechanical pattern due to factors such as fatigue (Radzak et al., 2017), previous injury (Zifchock et al., 2006) or various running techniques (Karamanidis et al., 2003). Comparatively, asymmetry in braking, or propulsive (anteroposterior) forces and resulting impulses, which could be important with regard to limb differences in contributing to maintenance of forward momentum, have rarely been explored. In one study of non-injured team sport players, no significant differences were found for any kinetic/kinematic variables between right and left legs (Brughelli et al., 2010). Only one running velocity (corresponding ~to 80% of maximum velocity of tested individuals) was explored, while any bilateral leg difference was not assessed using the recommended SA score (Exell et al., 2012a).

The main purpose of this study was to examine the effect of running velocity upon magnitude and range of asymmetry in the main kinetics and kinematics of treadmill running at constant, submaximal velocities.

## Methods

### Participants

Nine well-trained middle-distance runners (mean ± SD age, 26.5 ± 4.8 years; stature, 1.77 ± 0.05 m; body mass, 66.4 ± 4.2 kg; recent 5 km time, 15:58± 0:57 min:s) were recruited for this study. All participants had a minimum of 3 years consistent running training at a competitive level. Training volume for the 6 weeks preceding testing was 6–11 hr.wk^−1^ running, and 1–2 hr.wk^−1^ cross-training which involved a mixture of plyometrics, light resistance exercise, and run specific functional movement training. Foot strike pattern was determined using sagittal plane video-analysis at a sampling frequency of 240 Hz using an iPhone 7 (Apple, California, US). In our sample of nine participants, five and four were rearfoot and midsole strikers at 10 km.h^−1^, respectively. Participants had no known history of cardiovascular, neurological, or orthopedic problems, were injury free for the 3 months leading up to the data collection and gave written informed consent prior to participation in the study. Ethical approval for the study was provided by the Anti-Doping Laboratory Ethics Committee in Qatar (IRB Application Number: E2015000073) and was undertaken according to the principles outlined in the Declaration of Helsinki.

### Experimental Procedures

Testing took place during the competitive outdoor in-season (November) as part of a training camp in Doha (State of Qatar). The main experimental session started with the completion of a standardized warm-up (5 min of running at 10 km.h^−1^, followed by 1 min at 15 and 20 km.h^−1^ and 1–2 habituation runs of ~20 s at 25 km.h^−1^). After 5 min of passive rest, participants ran at seven running velocities (10, 12.5, 15, 17.5, 20, 22.5, and 25 km.h^−1^) for 60 s with >90 s of rest (quiet standing upright) between efforts. Running velocities were assigned in a randomized and counterbalanced order among participants. They ran on an instrumented treadmill (ADAL3D-WR, Medical Development—HEF Tecmachine, France) in an indoor facility maintained at standard environmental conditions (~24°C/45% of relative humidity). Participants commenced all rest-to-exercise transitions (or *vice-versa*) by holding the sidebars of the treadmill, while stepping directly on the moving treadmill belt during work intervals or on the sides of the treadmill during the recovery periods, respectively. They were asked to refrain from strenuous exercise, avoid caffeine and alcohol in the 24 h preceding the measurements, and to arrive at the testing sessions in a rested and hydrated state, at least 3 h postprandial. Participants confirmed that they were familiar with treadmill running and completed at least two training sessions on a treadmill in the year preceding the testing.

### Running Mechanics

Data were continuously sampled at 1,000 Hz, and after appropriate filtering (Butterworth-type 30 Hz low-pass filter, fourth order), instantaneous data of vertical, net horizontal, and total (resultant) ground reaction forces were averaged over the support phase of each step (vertical force above 30 N), and expressed in body weight (N). These data were completed by measurements of the main step kinematic variables: contact time (s), aerial time (s), step frequency (Hz), and step length (m). Peak braking and peak propulsive forces (body weight or BW), duration of braking and push-off phases (s) along with braking and push-off impulses (BW.s^−1^) were determined. Finally, vertical mean loading rate (BW.s^−1^) was calculated as the mean value of the time-derivate of vertical force signal within the first 50 ms of the support phase (Giandolini et al., 2013).

A linear spring-mass model of running was used to investigate the main mechanical integrative parameters characterizing the lower limbs behavior during running. Vertical stiffness (*K*_vert_ = F*z*_max_.Δz^−1^, kN.m^−1^) was calculated as the ratio of peak vertical forces (F*z*_max_ in N) to the maximal vertical downward displacement of center of mass (Δ*z* in m), which was determined by double integration of vertical acceleration of center of mass over time during ground contact. Leg stiffness (*K*_leg_ = F*z*_max_.Δ*L*^−1^, in kN.m^−1^) was calculated as the ratio of F*z*_max_ to the maximum leg spring compression (Δ*L*) [Δ*z* + L_0_-√(L_0_^2^–(0.5 × running velocity × contact time)^2^), in m], both occurring at mid-stance. Initial leg length (L_0_, great trochanter to ground distance in a standing position, in m) was determined from participant's stature as L_0_ = 0.53 × stature (Morin et al., 2005).

### Symmetry Angle

For each participant, inter-leg symmetry was measured using the symmetry angle (SA) and rectified so that all values were positive (Exell et al., 2012b). The SA was calculated using the equation below (Zifchock et al., 2008).

Symmetry angle (SA) =

$$\begin{array}{l} \frac{\left|\;45^{◦}\;-\;\left(\;tan^{-1}\;\left[\;\frac{left}{right}\;\right]\;\right)\;\right|}{90}\times 100\; \end{array}$$

but if

$$\begin{array}{l} \left(45^{◦}-tan^{-1}\left[\frac{left}{right}\right]\right)>90\; \end{array}$$

then

$$\begin{array}{l} \frac{\left|\;45^{◦}\;-\;\left(\;\tan^{-1}\;\left[\;\frac{left}{right}\;\right]-180\right)\;\right|}{90}\times 100\; \end{array}$$

The SA reports an absolute score (between 0 and 100%) that describes the deviation of the observed relationship between the two legs from a theoretically perfect relationship; where a score of 0% indicates perfect symmetry and 100% indicates perfect asymmetry.

### Data Analysis and Statistics

Ten consecutive steps (5 right and 5 leg foot contacts) beginning at the 50th second of each 60-s running bout were analyzed, and the averaged values were calculated for further analysis. In the context of our study (running velocities up to 25 km.h^−1^), this represents an optimal trade-off between enough time for gait to normalize for consistency of measurements and prevention of significant fatigue development that might otherwise influence running style. Participants were informed to “run normally” during the entire duration of each run, without knowing the exact moment of the sampling (Morin et al., 2009). Descriptive statistics are presented as mean values ± SD. Normal distribution of the data was checked by the Shapiro-Wilk normality test. Mechanical data were tested using a one factor (time) ANOVA for repeated measures (10, 12.5, 15, 17.5, 20, 22.5, and 25 km.h^−1^). To assess assumptions of variance, Mauchly's test of sphericity was performed using all ANOVA results. A Greenhouse-Geisser correction was performed to adjust the degree of freedom if an assumption was violated, while a Bonferroni *post-hoc* multiple comparison was performed if a significant main effect was observed. For each ANOVA, partial eta-squared (η^2^) was calculated as measures of effect size. Values of 0.01, 0.06, and above 0.14 were considered as small, medium and large, respectively. All statistical calculations were performed using SPSS statistical software V.24.0 (IBM Corp., Armonk, NY, USA). The significance level was set at *p* < 0.05.

## Results

**Figure 1:**
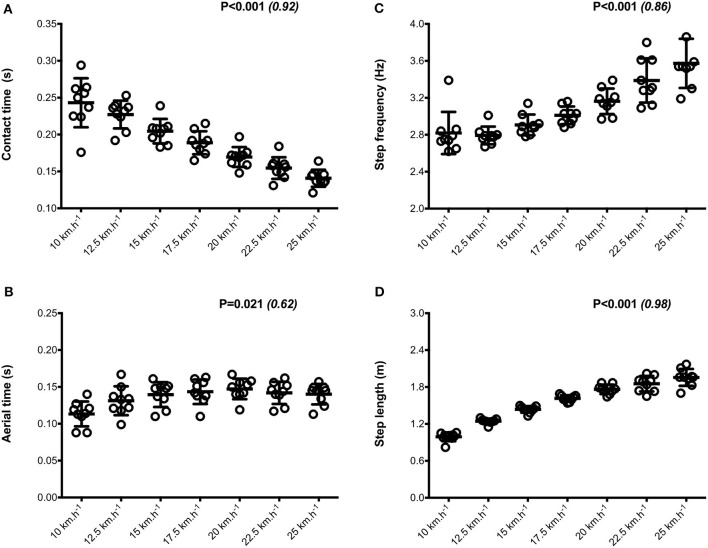
Spatio-temporal parameters. Contact time **(A)**; aerial time **(B)**; step frequency **(C)**; step length **(D)**. Values are mean ± SD. *P*-value and partial eta-squared in parentheses for the ANOVA main effect of running velocity.

**Figure 2:**
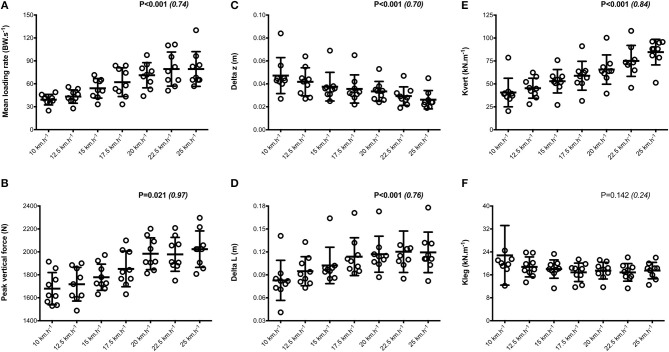
Dynamics and spring-mass variables. Mean loading rate **(A)**; peak vertical force **(B)**; Delta z, center of mass vertical displacement **(C)**; Delta L, leg compression **(D)**; Kleg, leg stiffness **(E)**; Kvert, vertical stiffness **(F)**. Values are mean ± SD. *P*-value and partial eta-squared in parentheses for the ANOVA main effect of running velocity.

**Figure 3:**
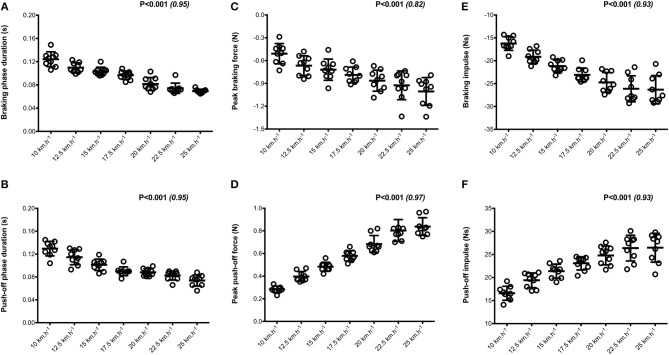
Horizontal force production variables. Braking and push-off phases duration (**A,B**, respectively); Peak braking and push-off forces (**C,D**, respectively); Braking and push-off impulses (**E,F**, respectively). Values are mean ± SD. *P*-value and partial eta-squared in parentheses for the ANOVA main effect of running velocity.

Mean SA scores for spatio-temporal variables were lower than 2%: contact time (0.6 ± 0.1%; range: 0.4–0.7%), aerial time (1.7 ± 0.2%; range: 1.3–2.1%) as well as step length and step frequency (0.7 ± 0.2%; range: 0.5–0.9%; Figure 1). Mean loading rate (5.3 ± 1.1%; range: 4.1–6.9%) and spring mass model [peak vertical force: 3.2 ± 1.6% (range: 2.9–3.4%); maximal downward vertical displacement: 11.2 ± 6.0% (range: 9.2–14.0%); leg compression: 3.6 ± 1.9% (range: 2.9–5.6%); vertical stiffness: 8.8 ± 1.9% (range: 7.1–11.6%); leg stiffness: 1.6 ± 0.6% (range: 1.2–2.9%)] presented a trend of larger mean SA scores (Figure 2). Mean SA scores ranged 1–4% for duration of braking (1.3 ± 0.3%; range: 0.9–2.0%) and push-off (1.6 ± 0.9%; range: 1.2–2.4%) phases, peak braking (2.4 ± 1.1%; range: 1.6–3.6%), and push-off (1.7 ± 0.9%; range: 1.2–2.2%) forces as well as braking (3.7 ± 2.0%; range: 2.8–5.8%) and push-off (2.1 ± 0.8%; range: 1.3–2.6%) impulses (Figure 3). However, with the exception of braking impulse (*P* = 0.005; η^2^ = 0.31), there was no influence of running velocity on mean SA scores for any of the mechanical variables studied (0.118<P<0.920; 0.01< η^2^ <0.23; Figures 1–3). Associations of SA scores between selected variables are displayed in Table 1.

**Table 1:** Relationships between symmetry angle scores of selected running mechanical variables (all running speeds combined; *n* = 9).

	**Contact time**	**Aerial time**	**Step frequency**	**Step length**	**Mean loading rate**	**Peak vertical force**	**Maximal downward vertical displacement**	**Leg compression**	**Vertical stiffness**	**Leg stiffness**	**Braking phase duration**	**Peak braking force**	**Braking impulse**	**Push–off phase duration**	**Peak push–off force**	**Push–off impulse**
Contact time	1	−0.092	−0.106	−0.106	0.189	−0.238	−0.147	−0.219	−0.124	0.240	0.086	−0.203	−0.226	0.523^**^	−0.156	0.194
Aerial time		1	0.858^**^	0.858^**^	0.003	0.247	0.471^**^	0.116	0.521^**^	−0.210	0.078	−0.267^*^	0.349^**^	0.083	0.269^*^	−0.060
Step frequency			1	1.000^**^	−0.038	0.110	0.351^**^	0.115	0.415^**^	−0.264^*^	0.166	−0.135	0.437^**^	0.010	0.389^**^	−0.039
Step length				1	−0.038	0.110	0.351^**^	0.115	0.415^**^	−0.264^*^	0.166	−0.135	0.437^**^	0.010	0.389^**^	−0.039
Mean loading rate					1	−0.152	−0.117	−0.222	−0.069	−0.007	0.079	−0.274^*^	−0.090	0.133	−0.052	−0.367^**^
Peak vertical forces						1	0.804^**^	0.624^**^	0.667^**^	0.204	−0.023	−0.045	0.139	−0.137	0.121	0.168
Maximal downward vertical displacement							1	0.651^**^	0.975^**^	0.123	−0.130	−0.164	0.118	−0.083	0.274^*^	0.235
Leg compression								1	0.594^**^	0.417^**^	0.023	0.058	0.328^**^	−0.215	0.185	0.248
Vertical stiffness									1	0.092	−0.141	−0.206	0.096	−0.047	0.304^*^	0.205
Leg stiffness										1	0.162	−0.016	−0.083	0.276^*^	−0.166	0.285^*^
Braking phase duration											1	0.197	0.387^**^	0.481^**^	−0.108	−0.047
Peak braking force												1	0.413^**^	−0.132	−0.064	0.214
Braking impulse													1	−0.075	0.180	−0.037
Push–off duration														1	−0.135	0.189
Peak push–off force															1	0.248
Push–off impulse																1

*Significant ANOVA P-values are represented in bold*.

* P < 0.05 and

** *P < 0.01 for significant relationships*.

**Figure 4:**
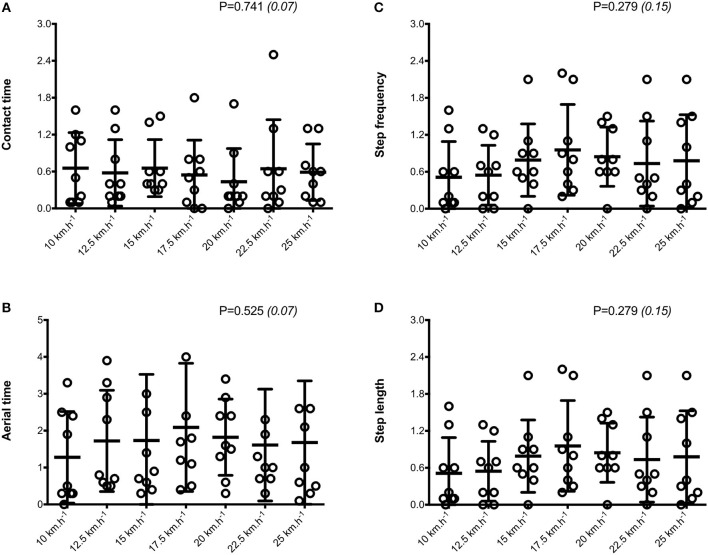
Symmetry angle scores (expressed as %) for spatio-temporal parameters. Contact time **(A)**; aerial time **(B)**; step frequency **(C)**; step length **(D)**. Values are mean ± SD. *P*-value and partial eta-squared in parentheses for the ANOVA main effect of running velocity.

**Figure 5:**
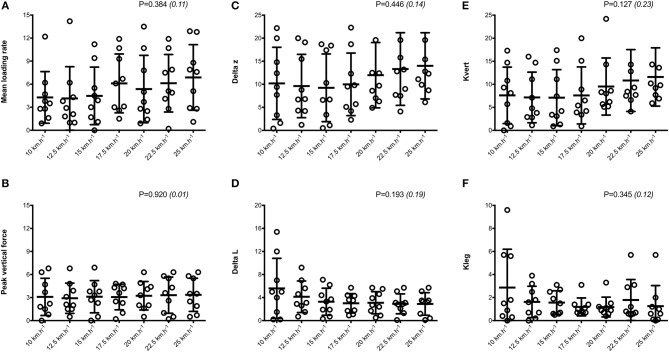
Symmetry angle scores (expressed as %) for dynamics and spring-mass variables. Mean loading rate **(A)**; peak vertical force **(B)**; Delta z, center of mass vertical displacement **(C)**; Delta L, leg compression **(D)**; Kleg, leg stiffness **(E)**; Kvert, vertical stiffness **(F)**. Values are mean ± SD. *P*-value and partial eta-squared in parentheses for the ANOVA main effect of running velocity.

**Figure 6:**
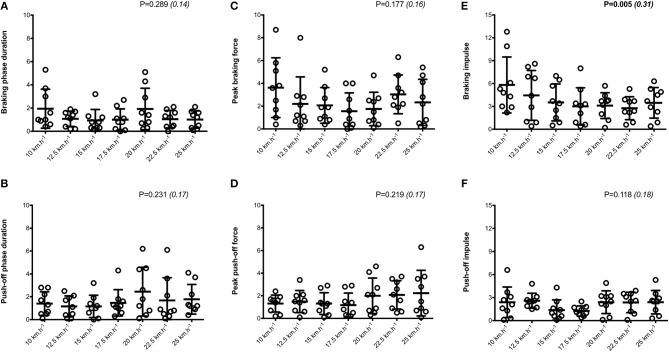
Symmetry angle scores (expressed as %) for horizontal force production variables. Braking and push-off phases duration (**A,B**, respectively); Peak braking and push-off forces (**C,D**, respectively); Braking and push-off impulses (**E,F**, respectively). Values are mean ± SD. *P*-value and partial eta-squared in parentheses for the ANOVA main effect of running velocity.

When all values were pooled for the both legs, increasing treadmill velocity from 10 to 25 km.h^−1^ induced shorter contact times (0.243 ± 0.033 vs. 0.141 ± 0.012 s; −41.2 ± 7.7%), longer aerial times (0.113 ± 0.017 vs. 0.140 ± 0.014 s; +25.6 ± 20.1%), faster step frequency (2.82 ± 0.23 vs. 3.59 ± 0.27 Hz; +27.2 ± 11.2%) along with longer step length (0.099 ± 0.071 vs. 0.195 ± 0.014 m; +98.0 ± 18.5%) (all *p* < 0.05; η^2^>0.62; Figure 4; Table 2). Values for mean loading rate (39.2 ± 6.8 vs. 79.4 ± 22.7 BW.s^−1^; +103.6 ± 45.0%) and vertical stiffness (40.6 ± 15.5 vs. 84.7 ± 13.9 kN.m^−1^; +122.4 ± 44.9%) all nearly doubled (all *p* < 0.001; η^2^>0.74), while changes in peak vertical force (1,679 ± 140 vs. 2,024 ± 159 N; +20.8 ± 7.7%), maximal downward vertical displacement (0.047 ± 0.016 vs. 0.026 ± 0.008 m; −43.0 ± 12.8%) and leg compression (0.083 ± 0.026 vs. 0.120 ± 0.026 m; +52.3 ± 46.9%) were more modest (*p* < 0.05; η^2^>0.70) and leg stiffness (22.8 ± 10.4 vs. 17.5 ± 2.8 kN.m^−1^; −16.2 ± 19.5%) remained unchanged (*P* = 0.142; η^2^ = 0.24; Figure 5; Table 3). Duration of braking (0.124 ± 0.013 vs. 0.069 ± 0.010 s; −43.8 ± 4.9%) and push-off (0.130 ± 0.013 vs. 0.074 ± 0.010 s; −43.0 ± 5.1%) phases shortened, while peak braking (−0.51 ± 0.14 vs. −1.01 ± 0.18 BW; +105.5 ± 43.5%) and push-off (0.28 ± 0.03 vs. 0.84 ± 0.08 BW; +197.5 ± 35.7%) forces as well as braking (−16.2 ± 1.5 vs. −26.3 ± 3.1 BW.s^−1^; +62.4 ± 17.3%) and push-off (16.6 ± 1.5 vs. 26.5 ± 3.1 BW.s^−1^; +60.3 ± 20.2%) impulses increased (*p* < 0.001; η^2^ > 0.82; Figure 6; Table 4).

**Table 2:** Relative changes for spatio–temporal parameters between 10 and 25 km.h^−1^ (*n* = 9).

**Variables (% change in reference to 10 km.h^**−1**^)**	**12.5 km.h^**−1**^**	**15 km.h^**−1**^**	**17.5 km.h^**−1**^**	**20 km.h^**−1**^**	**22.5 km.h^**−1**^**	**25 km.h^**−1**^**	***P*-value (Effect size)**
Contact time	−4.9 ± 16.0	−14.6 ± 13.0^a^^b^	−21.2 ± 10.8^a^^b^	−29.3 ± 10.1^a^^b^	−35.8 ± 6.8^a^^b^	−41.2 ± 7.7^a^^b^	<0.001 (0.92)
Aerial time	16.4 ± 13.3	23.9 ± 8.3^a^	27.3 ± 10.8^a^	31.3 ± 14.4^a^	26.8 ± 17.5^a^	25.6 ± 20.1^a^	<0.001 (0.62)
Step frequency	−0.5 ± 6.8	3.6 ± 7.6^b^	7.3 ± 7.2^b^	12.6 ± 7.9^a^^b^	20.3 ± 5.8^a^^b^	27.2 ± 11.2^a^	<0.001 (0.86)
Step length	26.1 ± 9.9^a^	45.6 ± 12.3^a^^b^	63.7 ± 12.6^a^^b^	78.5 ± 14.0^a^^b^	87.3 ± 8.9^a^	98.0 ± 18.5^a^	<0.001 (0.98)

*Values are mean ± SD*.

a *Significantly different from 10 km.h^−1^ (P < 0.05)*.

b *Significantly different from the previous running velocity (P < 0.05)*.

**Table 3:** Relative changes for dynamics and spring–mass variables between 10 and 25 km.h^−1^ (*n* = 9).

**Variables (% change in reference to 10 km.h^**−1**^)**	**12.5 km.h^**−1**^**	**15 km.h^**−1**^**	**17.5 km.h^**−1**^**	**20 km.h^**−1**^**	**22.5 km.h^**−1**^**	**25 km.h^**−1**^**	***P*-value (Effect size)**
Mean loading rate	10.2 ± 14.3	37.0 ± 15.0^a^^b^	57.4 ± 35.4^a^	81.0 ± 22.1^a^	101.3 ± 36.2^a^	103.6 ± 45.0^a^	**<0.001 (0.74)**
Peak vertical forces	2.7 ± 9.2	6.3 ± 7.6	10.5 ± 7.8	18.5 ± 8.2^a^^b^	18.4 ± 11.1^a^	20.8 ± 7.7^a^	**0.021 (0.97)**
Maximal downward vertical displacement	−9.0 ± 21.2	−20.1 ± 7.4^a^	−25.0 ± 4.9^a^	−27.6 ± 9.5^a^	−37.3 ± 7.1^a^^b^	−43.0 ± 12.8^a^	**<0.001 (0.70)**
Leg compression	22.0 ± 41.2	30.0 ± 38.0^a^	43.9 ± 37.5^a^	49.5 ± 43.9^a^	52.0 ± 39.4^a^	52.3 ± 46.9^a^	**<0.001 (0.76)**
Vertical stiffness	18.3 ± 28.9	34.6 ± 17.6^a^	48.7 ± 13.5^a^	67.6 ± 22.2^a^	92.7 ± 27.7^a^^b^	122.4 ± 44.9^a^	**<0.001 (0.84)**
Leg stiffness	−9.5 ± 23.4	−14.0 ± 18.8	−19.5 ± 18.1	−16.6 ± 18.5	−18.8 ± 20.2	−16.2 ± 19.5	0.142 (0.24)

*Values are mean ± SD*.

a *Significantly different from 10 km.h^−1^ (P < 0.05)*.

b *Significantly different from the previous running velocity (P < 0.05)*.

*Significant ANOVA P-values are represented in bold*.

**Table 4:** Relative changes for horizontal force production variables between 10 and 25 km.h^−1^ (*n* = 9).

**Variables (% change in reference to 10 km.h^**−1**^)**	**12.5 km.h^**−1**^**	**15 km.h^**−1**^**	**17.5 km.h^**−1**^**	**20 km.h^**−1**^**	**22.5 km.h^**−1**^**	**25 km.h^**−1**^**	***P*-value (Effect size)**
Braking phase duration	−11.4 ± 3.8^a^	−16.5 ± 4.8^a^^b^	−21.8 ± 5.5^a^^b^	−34.3 ± 5.7^a^^b^	−40.3 ± 3.9^a^	−43.8 ± 4.9^a^	<0.001 (0.95)
Peak braking force	33.8 ± 16.2^a^	44.2 ± 15.7^a^	61.0 ± 28.1^a^	77.8 ± 42.6^a^	89.9 ± 48.5^a^	105.5 ± 43.5^a^	<0.001 (0.82)
Braking impulse	18.0 ± 6.1	30.8 ± 8.4^a^^b^	42.7 ± 9.5^a^^b^	52.8 ± 13.5^a^^b^	61.5 ± 16.7^a^^b^	62.4 ± 17.3^a^	<0.001 (0.93)
Push–off phase duration	−11.4 ± 5.8^a^	−21.7 ± 4.0^a^^b^	−30.2 ± 6.9^a^^b^	−31.6 ± 7.0^a^	−36.2 ± 5.4^a^	−43.0 ± 5.1^a^	<0.001 (0.95)
Peak push–off force	39.7 ± 8.5^a^	70.4 ± 5.3^a^^b^	104.4 ± 11.2^a^^b^	141.6 ± 17.3^a^^b^	184.2 ± 29.9^a^^b^	197.5 ± 35.7^a^	<0.001 (0.97)
Push–off impulse	17.3 ± 8.6^a^	29.3 ± 10.0^a^^b^	39.9 ± 10.8^a^^b^	50.3 ± 14.8^a^^b^	59.8 ± 18.9^a^^b^	60.3 ± 20.2^a^	<0.001 (0.93)

*Values are mean ± SD*.

a *Significantly different from 10 km.h^−1^ (P < 0.05)*.

b *Significantly different from the previous running velocity (P < 0.05)*.

## Discussion

The purpose of our study was to examine kinematic and kinetic asymmetries, with specific interest in how these asymmetries change with increasing running velocity in uninjured runners. Runners' SA scores were analyzed at seven different velocities (range: 10–25 km.h^−1^) to determine whether a similar degree of asymmetry was present. Averaged SA values were small (<4%) across most spatio-temporal and spring mass variables, while horizontal force measures displayed larger asymmetry. However, there was virtually no influence of running velocity on asymmetry scores for any of the mechanical variables studied since our data exhibited relatively unchanging average values, and consistently low-to-moderate asymmetry scores across all velocities. Overall, asymmetries in all key mechanical parameters did not differ significantly between slower and faster running velocities.

### No Influence of Running Velocity on Asymmetry

Considerable evidence exists for natural kinetic asymmetries during both submaximal (Zifchock et al., 2008) and maximal velocity (Exell et al., 2012b) running. However, it is unclear whether or not the magnitude of these asymmetries changes with increasing velocity. Previously, imbalances in propulsion and maximal downward vertical displacement measures have been shown to increase with velocity (Belli et al., 1995), indicating a potential for greater asymmetry in running, while others found that an increase in running velocity does not fundamentally alter kinetic and kinematic symmetry (Karamanidis et al., 2003; Zifchock et al., 2006). In our study, with the exception of one variable (i.e., faster velocities had less asymmetry for braking impulse), we found no differences in SA scores across a range of slow and fast running velocities for >15 gait variables in non-injured, well trained runners. Even for spatiotemporal parameters, for which the average values change drastically, the level of asymmetry was consistently small across all velocities. Tested runners were also symmetrical for the ground reaction force variables measured, with minimal differences across a range of low-to-fast running velocities. The main observation of our study is that left and right asymmetry values of running kinetics and kinematics didn't increase as velocity varied between 10 and 25 km.h^−1^.

### Comparison of Asymmetry Between Variables

Although the important temporal variables of step frequency, step length, and contact time showed low asymmetry, SA scores for flight time were twice as large at all velocities, as also reported elsewhere (Karamanidis et al., 2003). In general, SA scores for horizontal force production parameters including braking and push-off phase durations, peak forces, and impulses were similar to those measured for spatiotemporal variables. Minimal differences between limbs during running across a wide range of velocities suggest that limbs may not be used preferentially for braking or propulsion. However, mean SA scores above 3% were found for mean loading rate and spring mass model variables for all velocities, as also observed when running at 16 km.h^−1^ (Pappas et al., 2015), indicating that asymmetry increases in variables derived from the vertical force signal. Rumpf et al. (2014) also showed that asymmetries in vertical force (~20%) are significantly greater than those of the horizontal force (~15%) during a 30-m sprint on a non-motorized force treadmill. In contrast, up to three times larger deviations from symmetry were detected for mean horizontal compared to vertical forces during maximal treadmill sprinting, during both the early (Brown et al., 2017) and late (Girard et al., 2017a) acceleration phases. In this study using a wide range of running velocities, kinetic asymmetries tended to be larger than temporal ones at the fastest velocities, a finding also reported elsewhere (Exell et al., 2012a). A qualitative inspection of the average bilateral asymmetries further indicates that maximal downward vertical displacement was the only mechanical variable exceeding 10% (i.e., 11.2 ± 6.0%) across velocities. Anecdotally, our unique set of data establishes preliminary normative data for describing patterns of lateralization across a wide range of running velocities and for a large number of mechanical variables in non-injured individuals. However, there is no definitive answer as to which level asymmetries really describe true differences between limbs. Whereas some groups have considered inter-limb differences significant only if the asymmetry score is >10% (Zifchock et al., 2006, 2008), others suggest that asymmetry must exceed intra-limb variability to be considered significant (Exell et al., 2012b). These data reinforce the notion that asymmetry is task- and variable-dependent and highlight limitations in applying arbitrary thresholds to determine acceptable between-limb differences.

### Asymmetry Scores and Between-Participant's Variability

Whereas key gait variables displayed symmetrical gait overall, the individual nature of asymmetry was demonstrated by the relatively large range of asymmetry between participants for some variables (e.g., ~0–10 and ~0–20% for leg stiffness and vertical stiffness, respectively), across the range of velocities tested. This reinforces the importance of assessing asymmetry on an individual basis rather than using group means as athletes use different mechanisms for contralateral limbs to achieve similar outcomes (Exell et al., 2012b). Our SA data also demonstrate that patterns of change across different velocities are consistent between limbs for some but not all parameters, as shown by low-to-moderate correlations coefficients between selected variables. In general, significant relationships were observed between variables that are intrinsically linked; i.e., aerial time with step frequency (*r* = 0.86; *p* < 0.01) and either peak vertical force (*r* = 0.67 *p* < 0.01) or maximal downward vertical displacement (*r*– = 0.98; *p* < 0.01) with vertical stiffness. Testing asymmetry during maximal sprint performance in 11–16 year old boys, Meyers et al. (2017) reported weak yet significant relationships (*r* = −0.24 to 0.39; *p* < 0.05) between sprint velocity and a variety of asymmetry metrics including step frequency, step length, flight time, and vertical stiffness. The results of the current study also only partially agree with those of Belli et al. (1995). Indeed, the highest standard deviations exhibited by the kinetic (e.g., maximal downward vertical displacement, leg stiffness) and kinematic (e.g., contact time, braking phase duration) parameters were not always observed at the fastest velocity. Athletes and their support staff should be careful not to infer the presence of asymmetry based on a single or limited number of measurements given the variable nature of asymmetry evident in our study and those of others (Bishop et al., 2019).

### Changes in Running Mechanical Variables Across Velocities

Our entire set of mechanical data and the trends for changes in the main biomechanical variables are within the range of those encountered in the literature for similar external conditions. For example, from 10 to 20 km.h^−1^ stride length increased by 78.5% and stride frequency by 12.6%. These values are comparable in magnitude to those reported elsewhere (~77 and ~17%, respectively) for team-sport athletes running at similar velocities on the same instrumented treadmill (Girard et al., 2017b). This confirms that running velocity increases mainly by lengthening stride in the range of submaximal velocities tested here, while stride frequency becomes more important at faster velocities (> 25 km.h^−1^) (Brughelli et al., 2011) and that is also associated with a 2-fold increase in vertical loading rates (Breine et al., 2014). Furthermore, our data displayed a linear reduction in contact time from the slowest to fastest velocities, while aerial time lengthened from 10 to 15 km^−1^ with no meaningful change thereafter. Similar to previous studies (Brughelli et al., 2011), peak vertical forces remained relatively constant when velocity increased from 20 km.h^−1^ and beyond, while there was no plateau of either peak braking force or push-off force both increasing linearly with velocity. We add the interesting observation that pushing more rather than braking less (in terms of peak forces) is the natural strategy used to increase running velocity from 10 to 25 km.h^−1^. Indeed, with treadmill velocity increase, the magnitude of change for peak push-off force was twice as large as peak braking force, yet with similar corresponding decrements in braking and push-off phase durations. While braking time does not decrease nearly as much as push time as velocity increases from <5 km.h^−1^ to > 20 km.h^−1^ (Cavagna, 2006), durations of the two phases in the range 10–20 km.h^−1^ is relatively similar.

By modeling the lower limb as a linear mass-spring system, mechanical spring constants can be used to describe the resistance of either center of mass vertical displacement or leg compression to a corresponding vertical force, known as vertical and leg stiffness, respectively (Brughelli and Cronin, 2008). Whereas, leg stiffness modifications were not significant across the range of velocities tested here, vertical stiffness was the spring mass model variable that displayed larger relative changes. Interestingly, the relative increase in peak vertical force only represented half of the decrease in maximal downward vertical displacement. These changes were almost identical to those of other trained runners tested at similar treadmill velocities (Brughelli et al., 2011). As velocity increases, higher stiffness values indicate that the center of mass undergoes smaller vertical displacement when confronted with an applied vertical force.

### Limitations and Additional Considerations

In the present study we only examined nine healthy runners, which may limit the generalization of our results to a broader (i.e., previously injured) population, even though we found moderate-to-large effect sizes in general. Marked asymmetry in vertical force (but not contact times) during running in ACL reconstructed soccer players exist <9 months post-surgery, with these asymmetries also appeared to slightly increase with increasing speed, despite meeting functional criteria for return to sport (Thomson et al., 2018). Using a larger sample, it might be useful to examine if measures of asymmetries are evident more superiorly in rear-foot (as most marathon runners during the 2017 IAAF World Championships; Hanley et al., 2019) compared to forefoot or mid-foot strikers. Second, it cannot be ruled out that treadmill running may have artificially masked gait asymmetry. Treadmill compared to overground running that prevent any conscious or unconscious targeting of the force plates by the participants (the sampling effect), and thereby produce inherently low movement variability, also leads to improvement in SA scores at least in patients with knee osteoarthritis (Robadey et al., 2018). Using a treadmill allows for running velocity to be strictly controlled and maintained compared to outdoor running. However, most runners prefer running overground over running on a treadmill, especially when running at faster velocity, as this practice often is perceived as less comfortable (Miller et al., 2019). Perhaps the adaptation of our participants to treadmill running was to increase stride length at the higher velocities as opposed to stride frequency. Foot-worn inertial sensors for reliable detection of running gait temporal events (stride temporal parameters) may be useful to verify these assumptions and offer direct comparisons with data obtained under ecological situations (Falbriard et al., 2018). Third, it remains of particular interest to determine how gait asymmetries are influenced by the type of ground and how the effects of various external factors [i.e., fatigue (Radzak et al., 2017), footwear (Vagenas and Hoshizaki, 1992), training experience (Cavagna et al., 1977), limb dominance (Potdevin et al., 2008), and/or gait retraining with real-time feedback (Dingwell et al., 1996)] may be dependent on the running velocity at which they are assessed. In particular, the effect of footwear with different midsole thickness on SA scores in relation to running velocity changes warrants further consideration since this has the potential to influence running mechanics (Hamill and Gruber, 2017). Finally, we did not measure lower limb joint kinematics in this study and thus the effect of running velocity on the contribution that is made by individual joints (and potential neuro-mechanical compensations between joints; Brughelli and Cronin, 2008) is unclear.

## Conclusion

The results of the current study showed large variability in SA scores across running mechanical parameters, and these values are largely unaffected by increasing running velocity from 10 to 25 km.h^−1^. These data indicate that faster running velocities have no meaningful influence on the sensitivity of detecting gait asymmetries in non-injured, well trained runners.

## Data Availability Statement

The datasets generated for this study are available on request to the corresponding author.

## Ethics Statement

This studies involving human participants were reviewed and approved by Anti-Doping Laboratory Ethics Committee in Qatar (IRB Application Number: E2015000073). The patients/participants provided their written informed consent to participate in this study.

## Author Contributions

OG and NT conceived and designed the study. OG, PR, and NT conducted the experiments. All authors analyzed the data and drafted the manuscript, approved the manuscript.

### Conflict of Interest

The authors declare that the research was conducted in the absence of any commercial or financial relationships that could be construed as a potential conflict of interest.
